# Rational approach to the prescription of anti-rheumatic drugs in rheumatoid arthritis: a product leaflet-based strategy in Italy

**DOI:** 10.3389/fimmu.2024.1398314

**Published:** 2024-06-24

**Authors:** Carlo Perricone, Andrea Castellucci, Giacomo Cafaro, Santina Calvacchi, Lorenza Bruno, Roberto Dal Pozzolo, Francesco Tromby, Anna Colangelo, Roberto Gerli, Elena Bartoloni

**Affiliations:** Rheumatology, Department of Medicine and Surgery, University of Perugia, Perugia, Italy

**Keywords:** recommendations, EULAR, rheumatoid arthritis, treatment, DMARDs, JAKi, product leaflet

## Abstract

The treatment of patients with rheumatoid arthritis (RA) has dramatically changed in the past 30 years. Currently, numerous conventional, biologic, and targeted synthetic DMARDs have been licensed and used following recommendations provided by international and national scientific societies. However, the availability of biosimilars and the increasing necessity of savings impacted on the local/national prescription of these drugs. The information provided by data sheet of every single drug is a decisive factor on the choice of a certain treatment merged with the patient’s profile. Thus, our purpose was to construct a rational algorithm for the treatment strategy in RA according to costs and the product leaflet of the biologic and targeted-synthetic DMARDs currently licensed in Italy. We used the most recent available recommendations and then we performed a review of the literature considering all the factors that are known to influence drug safety/effectiveness. All these factors were considered in the context of the data sheets of currently available originators and biosimilars.

## Introduction

Improvements in the control of inflammation in rheumatoid arthritis (RA) by conventional synthetic and biologic disease-modifying antirheumatic drugs (DMARDs) have led to a substantial change in the clinical outcomes of patients during the last 30 years. Currently, multiple DMARDs have been licensed for the treatment of this disease, including conventional synthetic (cs) DMARDs (methotrexate (MTX), leflunomide, sulfasalazine); glucocorticoids (GCs); biological (b) DMARDs [adalimumab (ADA) (1), certolizumab pegol (CZP) (2), etanercept (ETN) (3), golimumab (GOL) (4), infliximab (IFX) (5), abatacept (ABA) (6), rituximab (RTX) (7), tocilizumab (TCZ) (8), sarilumab (SAR) (9), and their biosimilar DMARDs], and targeted synthetic (ts) DMARDs [baricitinib (BARI) (10), filgotinib (FILGO) (11), tofacitinib (TOFA) (12), upadacitinib (UPA) (13)]. Numerous randomized clinical trials provided relevant results on the efficacy and safety of these drugs both in monotherapy and in combination with csDMARDs, but in routine clinical practice an all-round algorithm based on scientific evidence and licensed indications may be of great help in building a treatment strategy as close as possible to the concept of tailored therapy. Several recommendations/guidelines, including those of the American College of Rheumatology (ACR) (14), European League Against Rheumatism (EULAR) (15) and of National scientific Societies from different countries including the Italian Society of Rheumatology (Società Italiana di Reumatologia - SIR) (16), provide general guidance in the identification of patient subsets that would benefit from a specific treatment. However, these recommendations do not provide a deep focus on single patient characteristics. For instance, the fact that some drugs cannot be used due to their potential to trigger an allergic reaction or cannot be used in patients with specific metabolic diseases is not mentioned. Furthermore, the use of an algorithm based on product leaflets may also have a legal implication in patients experiencing adverse reactions (17). Indeed, the information provided in the data sheets of every single drug is a decisive factor on the choice of a certain treatment over another with respect to the patient’s profile. It is noteworthy to underline that some information contained in the product leaflets can be obsolete at the light of more recent literature [for instance the case of concomitant use of ETN and sulfasalazine that was found to have an increased risk of reduced white blood cells count (18), a data later not confirmed (19)]. This will be detailed in all situations.

Thus, we aimed to provide a rational and complete treatment strategy algorithm for RA patients, based on the most recent developments in the field and accounting for drugs data sheets. At all circumstances the impact of economic factors was considered, and we applied a general principle following EULAR Task Force’s view on costs: if two drugs are equally appropriate for a specific patient, then the drug that is less costly should be used (15).

## Methods

The available product leaflets of the bDMARDs and tsDMARDs currently licensed in Italy for RA were reviewed up to the 31^st^ of December 2022. The drugs that were evaluated include ABA, ADA, BARI, CZP, ETN, FILGO, GOL, IFX, RTX, SAR, TCZ, TOFA, UPA and the available biosimilars. We did not provide data on the abovementioned drugs at dosages different to those approved in RA. Notably, anakinra was not included, given the relatively low efficacy of this agent except probably in patients with type 2 diabetes, compared to other b- and tsDMARDs, without a significant difference in pricing (20, 21)

Next, we pursued to follow the indications provided by the EULAR recommendations (15) on the treatment of RA. Then, we ranked the drugs according to mean pricing of these drugs in Italy (from the cheapest to the most expensive at the time of reviewing, according to prices in Regione Umbria). To drive the algorithm, we searched for all the possible side effects of the drugs, and we ranked these drugs according to contraindications, special warnings and precautions for use in specific clinical scenarios. Finally, an expert panel including specialists in rheumatology was constituted to review the literature on the existing evidence on the different variables influencing the biologic choice in patient with RA. Other aspects were considered as well, such as disease severity, efficacy and safety, monotherapy, response predictors including biomarkers, extra-articular manifestations, comorbidities, fertility, childbearing potential, pregnancy, infection, latent tuberculosis infection (LTBI) reactivation, cardiovascular and malignancy risk, interval and route of administration, patient’s preference, factors influencing the adherence to therapy.

Thus, we provided the first-line preferred options and the possible alternatives as second-line options. We used the definition “caution” when the risk/benefit ratio should be carefully evaluated, and possible alternatives should be considered. We used the term “avoid” when the drug is contraindicated, should not be used according to the product data sheets and the usage should be considered as off-label. In order to provide an immediate visual summary, decision trees were built.

## Results

**Figure 1:**
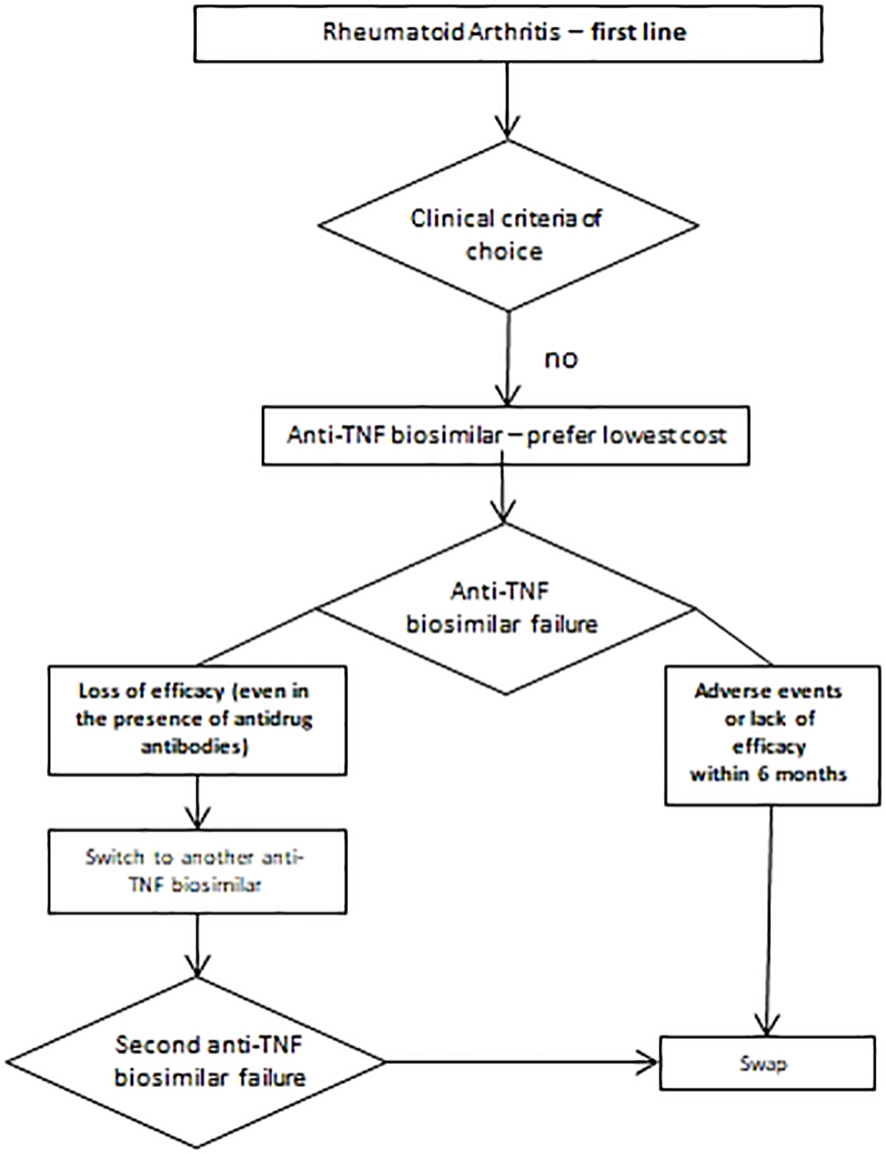
Therapeutic approach in patients without selection criteria.

In absence of head-to-head trials, the efficacy of the anti-TNF ADA, CZP, ETN, GOL, IFX and the available biosimilars for the treatment of RA has been evaluated by indirect comparison in several systematic reviews and meta-analyses (22). No significant differences have emerged among these drugs. The cheapest drugs so far are adalimumab biosimilars, to be chosen as first-line choice in the absence of other criteria (**Figure 1**).

### Risk of infections

Patients with RA are at higher risk of infections and the use of bDMARDS and tsDMARDS can increase such risk (23, 24). Older age, disease duration, disease activity, type of treatment and the association with glucocorticoids may have an impact on the augmented risk of developing a serious infection in RA patients (25). In patients with high-risk of infection, anti-TNF seem to be reasonably safe and in particular, as first-line therapy, ETN confirms a trend of lower risk of infections (3), while ABA can be suggested as a second line therapy (26). Indeed, it was found that in patients previously hospitalized for infection while receiving anti-TNF agents, both ABA an ETN have a lower risk of subsequent infection compared to other biologics (27). Nonetheless, in all patients suffering from severe infections including sepsis, abscesses, and opportunistic infections, the administration of any of b- or tsDMARD is contraindicated. FILGO, TOFA and UPA should not be used, or should be used with caution, in patients who have lived in or traveled to areas with endemic mycoses (11–13, 28). BARI should be preferably given at 2 mg/day in patients with chronic or recurrent infections (10). At the same extent, besides not specified in the product leaflet, it would be better to use Filgotinib at 100 mg/day in patients with an infectious risk (29).

LTBI is part of the recommended screening for all the considered drugs. Despite in patients from registry it was shown that the incidence of tuberculosis appears higher among users of anti-TNF than among users of other non-anti-TNF biologics (30), anti-TNF drugs can be employed with a satisfactory safety profile in patients with risk of LTBI (31). among anti-TNF, a trend towards a better safety profile of ETN in terms of infection and TB risk resulted from systematic reviews, meta-analyses, and national registries of patients treated with biologics (32). TCZ, ABA and JAKi may represent a valid second-line treatment option (33).

Regarding HBV infections, the SIR recently provided recommendations for the management of patients with RA (34). In general, medical literature offers evidence that TNF synergizes with interferon (35), and since the action of these molecules is extremely important in clearing the infection, it is mandatory to screen patients before starting anti-TNF agents. If the infection is found to be active, it is important to use antiviral treatment, possibly a month prior to the start of the anti-TNF and avoiding the use of RTX (7). In patients with non-active infection with occult HBV infection (HBV carriers) treated with RTX, patients should be carefully monitored and it is be advisable to consider prophylaxis with lamivudine when monitoring is not guaranteed (36). During chronic HBV infection the first-line choice falls on ADA (1) and IFX (5).

As far as Hepatitis C virus (HCV) infection reactivation is concerned, there appears to be no significant difference between bDMARDs and tsDMARDs (37), both appearing to be relatively safe for treatment of RA patients. The exception is represented by RTX that, despite being used to treat some HCV-related manifestations, such as cryoglobulinemia (38), may significantly increase HCV viral load (39). Thus, this treatment should be administered preferably in combination with antiviral therapy for HCV.

**Figure 2:**
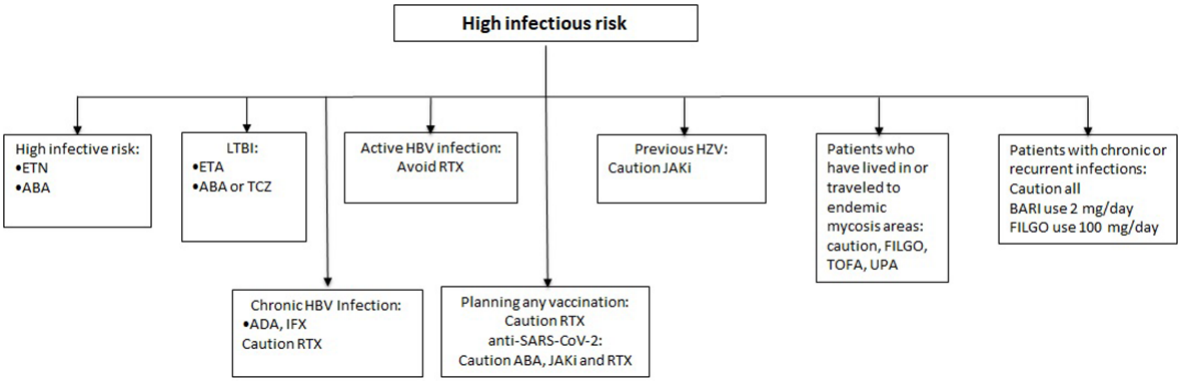
Therapeutic approach in patients with infectious risk.

Even though the potential association between Herpes Zoster (HZV) reactivation and immunosuppressive therapy is not clear, the evidence suggests to be aware of a potential higher risk with the use of the JAK inhibitors (12–15, 40) and a caution with the use of SAR (9, 41). Nonetheless, all the product leaflets require screening for TB, HBV and HCV prior to the beginning of any b- or tsDMARDs, underlining a certain carefulness in these conditions, need of patients’ monitoring and eventual use of concomitant agents for the treatment of the infections. Finally, it is important to adequately schedule the recommended vaccinations, especially in patients starting RTX treatment (42). ETN, CZP, IFX, TCZ, SAR, TOFA, BARI and UPA should not be administered in patients receiving live attenuated vaccines. ETN, TCZ, SAR, BARI, FILGO and UPA should be avoided also in case of administration of live vaccines. More recently, evidence of an impaired immunization after anti-SARS-CoV-2 vaccination has been found for RTX, ABA, and JAKi (43). Eventually, vaccines can be administered concomitantly, except for live vaccines in patients treated with ADA or GOL. A summary of the proposed algorithm for patients with high-risk of infection is shown in **Figure 2**.

### Pregnancy

Given that maternal immunoglobulins can pass through the placental barrier by selectively binding to the neonatal Fc receptor (44, 45), treatment with biological drugs during pregnancy must be carefully considered. If we should consider product leaflets only, pregnancy should be avoided for all the considered drugs, basically due to lack of consistent data or potential teratogenic risk. Maternal treatment with anti-TNF drugs after gestational week 30 can lead to fetal serum drug levels equal or higher than maternal ones, with potential complications, such as a slightly increased rate of birth defects, a significantly lower birth weight and a higher rate of preterm births (46).

However, among biologics, TNF-inhibitors are the most studied and appear reasonably safe to administer in the first to second trimester (47). CZP is employed as first-line therapy in pregnant women due to very limited levels of drugs detectable in fetal blood, but still it should be used only of clinically necessary (2). CZP is not actively transported across the placenta during pregnancy, but the Fab fragment may passively cross the placenta at low levels during the first trimester. Drug concentrations in the cord blood and in the infant at birth have been evaluated in 31 pregnancies exposed to IFX, ADA, and CZP. At birth, the median levels of IFX, ADA, and CZP compared with the concentration in maternal blood were 160%, 153%, and 3.9%, respectively. IFX and ADA could be detected in the infants for as long as 6 months (48). ETN can be used as a second-line option since it appears to be safer compared to other anti-TNF (3, 48). BARI, FILGO, TOFA and UPA have a short half-life and can be interrupted before pregnancy (10–13). Thus, they can be useful for couples who are willing to plan their pregnancy. Women should use a highly effective contraceptive during JAKi treatment. In the case of BARI and FILGO, the contraceptive measures should be used up to 1 week after the end of the treatment, this period should be extended to 4 weeks in patients treated with TOFA and UPA. Other drugs are preferably avoided. The evidence on the safety of ABA and RTX during pregnancy is very scarce. ABA does not seem to increase the risk of abortion. However, an increased rate of congenital anomalies cannot be excluded. Notably, Kumar et al. (49) did not confirm such report, the authors found no cases of vertebral defects, anal atresia, cardiac defects, tracheo-esophageal fistulas, renal anomalies, or limb abnormalities and an expected range of spontaneous abortion. An increased rate of spontaneous abortions and prematurity have been reported with RTX, along with mild and transient neutropenia and B-cell depletion in 12% of the new-borns (even if there are no reports of neonatal deaths or congenital malformations). Hence, it is not advisable to use this drug during pregnancy (7, 50–52). There are no data on the use of SAR in pregnancy, thus this drug should be used only if no better option is available and fertile women should take contraceptive measures during and up to 3 months to the end of the treatment (53).

**Figure 3:**
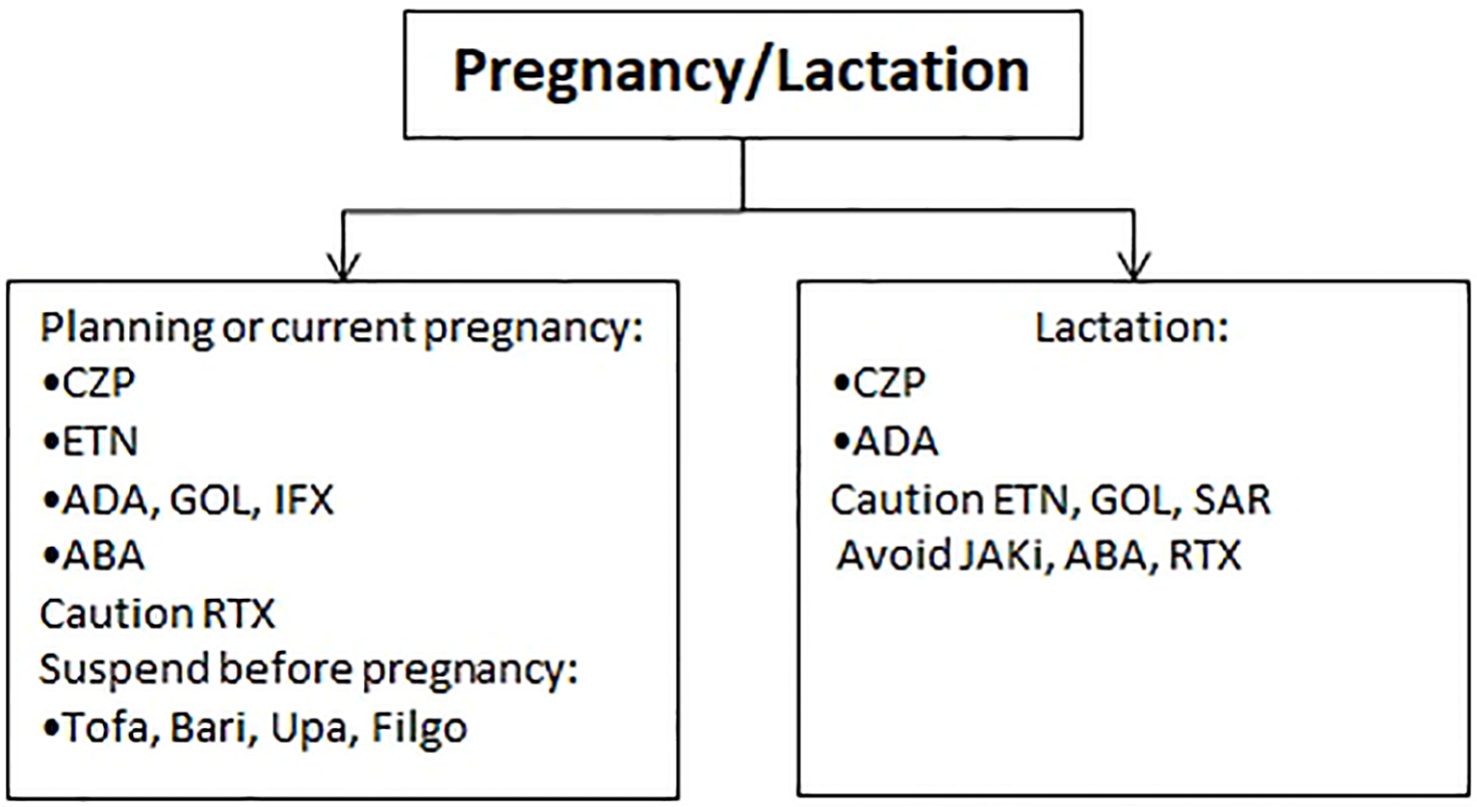
Therapeutic approach during pregnancy and lactation.

Studies on drug excretion into human breast milk are rare and mostly based on single-dose or short-term treatment, therefore grading of evidence for all drugs is ‘very low’ (53). The first-line therapy option is still CZP (2), followed by ADA (1) as second-line. Indeed, in a clinical study involving 17 women receiving CZP while breastfeeding, there was a minimal transfer of the drug from plasma to breast milk (54). The percentage of maternal dose of CZP reaching an infant in a 24-hour period was estimated from 0.04% to 0.3% (2). Also, given that CZP is degraded in the gastrointestinal tract after oral administration, the absolute bioavailability is considered very low in a breastfed infant. The use of ETN, GOL, SAR, is not recommended, while the latest scientific evidence contraindicates the use of BARI, FILGO, TOFA, UPA, ABA and RTX (55, 56) (**Figure 3**).

### Dysmetabolic and cardiovascular comorbidity

Autoimmune and inflammatory diseases such as RA are related to significantly higher risk of cardiovascular diseases (CVD) compared to general population and CVD are the leading cause of death in these patients (57–59). Therefore, it is exceptionally important to minimize modifiable risk factors, to reduce the degree of inflammation due to disease activity and to carefully consider all these aspects when selecting a treatment strategy (57). With specific reference to patients with high CV risk, anti-TNF drugs are considered fairly safe: altogether, there are several studies that show how anti-TNF therapy was significantly associated with a reduction in the risk of all CV events in RA (60–62). Therefore ETN and ADA can be employed as first line therapy in these situations (1, 3), followed by TCZ and ABA (6, 8). The CV risk related to intravenous infusion (thus concerning IFX, TCZ, ABA and RTX) should be considered, especially modulating infusion speed and avoiding intravenous route in patients with significant cardiovascular diseases including arrhythmias. Indeed, low, or elevated blood pressure can occur in up to 18.8% of patients treated with RTX in monotherapy (7).

In congestive heart failure (CHF) at level I and II of the New York Heart Association (NYHA) scale, medical literature and the drugs data sheets suggest to preferentially avoid the use of ADA, IFX, CZP and GOL (1, 2, 4, 5, 63); in patients with more severe CHF (NYHA III and IV), TCZ, SAR, ABA or a JAKi should be preferred as first-line agents (6, 8, 9, 63). Unlike milder cases of CHF, ETN is also not recommended (3, 64). In fact, post-marketing cases of CHF worsening, with or without identifiable precipitating factors, have been reported in patients treated with ETN. Rare cases (<0.1%) of new-onset CHF, including CHF in patients without pre-existing cardiovascular disease, have been reported too (65). Some of these patients were younger than 50 years of age. Intuitively, IFX, ADA, GOL, CZP are contraindicated as well (1, 2, 4, 5).

In patients with a history of deep vein thrombosis (DVT), pulmonary embolism or any hereditary bleeding disorders, there is no clear evidence on what the best drug to use is. However, the use of BARI, FILGO, TOFA and UPA appears to require caution (10–13, 66).

Furthermore, an *ad hoc* trial comparing the combined TOFA doses with a TNF inhibitor in a cardiovascular risk-enriched population, highlighted the possibility that the risk of major adverse cardiovascular events (MACE) and cancers was higher with TOFA, as non-inferiority criteria was not achieved (67). This led to a recommendation provided by the European Medicines Agency (EMA) that endorsed the measures recommended by the Pharmacovigilance Risk Assessment Committee (PRAC) to minimize risk of serious side effects with JAKi for chronic inflammatory disorders stating that “these medicines should be used in the following patients only if no suitable treatment alternatives are available: those aged 65 years or above, those at increased risk of major cardiovascular problems (such as heart attack or stroke), those who smoke or have done so for a long time in the past and those at increased risk of cancer.” JAK inhibitors should be used with caution in patients with risk factors for blood clots in the lungs and in deep veins (venous thromboembolism, VTE) other than those listed above. Further, the doses should be reduced in patient groups who are at risk of VTE, cancer or major cardiovascular problems, where possible” (68).

**Figure 4:**
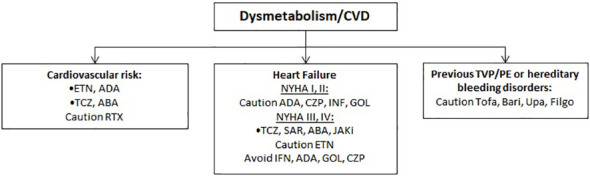
Therapeutic approach in patients with cardiovascular risk.

Of note, data from clinical trials as well as from real life studies seem to reassure on this risk (69, 70). Nonetheless, the Italian Medicines Agency (AIFA) released a note emphasizing the higher cardiovascular and cancer risk detected in patients using JAKi due to a supposed “class effect” of these drugs (71) (**Figure 4**).

### Comorbidities

The relative deficiency of TNF caused by anti-TNF therapy can lead to the initiation of an autoimmune process (72). In patients with a clear systemic autoimmune disease or a risk of Lupus-like disease (ANA ≥ 1:80), drugs as ABA, TCZ, SAR, RTX or JAKi should be considered as first-line therapy (6–9). Because the use of anti-TNF has some risk, it should be considered with caution. Specifically, a higher proportion of patients treated with IFX, ADA, ETN, GOL and CZP may develop ANA>1:40, anti-dsDNA and other autoantibodies compared with placebo. In some patients, including those with positive rheumatoid factor, a lupus-like syndrome or skin reactions clinically and at pathology, compatible with subacute cutaneous lupus or discoid lupus, can occur (73, 74). Thus, the new finding of anti-dsDNA antibodies must lead to anti-TNF drug discontinuation (75).

Interstitial lung disease (ILD) during RA is a quite frequent complication that could be dealt with during the course of this disease and it is a leading cause of death in patients with RA, associated with significant morbidity and mortality (76). ABA, RTX and TCZ are the most appropriate drugs as first-line agents (6–8, 77, 78). Nonetheless, the pre-existence of RA-related ILD may cause a higher risk of infection during treatment with TCZ and TOFA.

Chronic obstructive pulmonary disease (COPD) is a quite frequent comorbidity in RA patients. The use of ADA, IFX, CZP and GOL, especially in patients with moderate to severe COPD and heavy smokers, may be related to an increased risk of malignant tumors as found in a study on IFN (79). Noteworthy, ETN leaflet does not provide any recommendation on this and further studies on other anti-TNF did not confirm those preliminary observations (80). Some reports suggest an increased risk of exacerbations and dyspnoea in COPD patients treated with ABA (1, 2, 4–6, 81).

Some biological drugs, especially anti-TNFα, may increase the risk of developing demyelinating diseases (82, 83). In addition, rare reports of demyelinating polyneuropathies have been published (including Guillain-Barré syndrome, inflammatory demyelinating polyneuropathy, demyelinating polyneuropathy and multi focal motor neuropathy). Patients with multiple sclerosis may suffer from increased disease activity. For these reasons, anti-TNF drugs are contraindicated in patients with demyelinating diseases and a neurological examination should be performed to assess the benefit to risk ratio (1–6). Due to a well-known association between intermediate uveitis and central nervous system demyelinating diseases, it is recommended to perform a neurological evaluation in patients with non-infectious intermediate uveitis before initiating ADA therapy and at regular intervals. ABA, TCZ, RTX, SAR, JAKi can be employed as first-line therapy, having instead a fairly safe therapeutic profile in this context. ADA and IFX can be used as first-line therapy (1, 5, 84) in acute anterior uveitis, bearing in mind that ETN appears to be relatively contraindicated in this case (3, 85). Indeed, besides data from the product leaflet suggests that uveitis can occur at higher rates in patients treated with ETN, data from the literature suggest that ETN has a lower risk of developing uveitis compared to placebo (86) and in certain cases can be used to treat uveitis (87).

**Figure 5:**
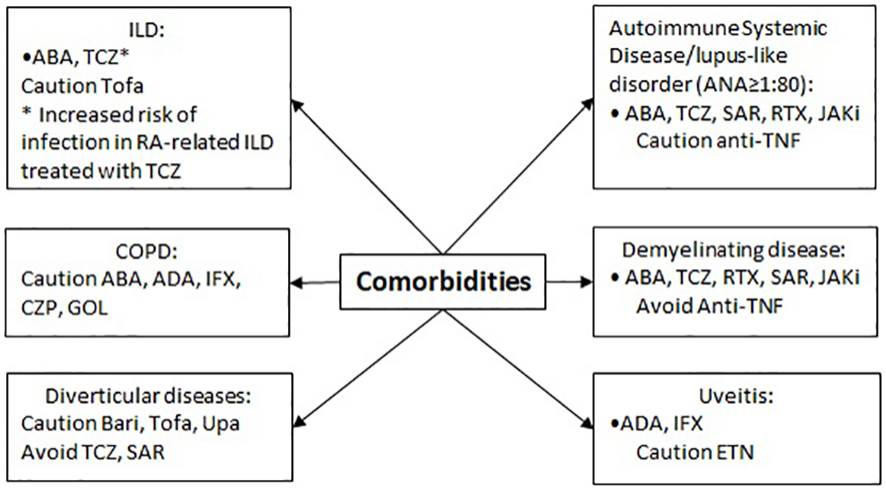
Therapeutic approach in patients with other comorbidities.

The existence of a diverticular disease or of a condition predisposing to gastrointestinal perforation requires a certain caution in the use of BARI, TOFA and UPA (10, 12, 13), while TCZ and SAR should be avoided (8, 9), due to an increased risk of diverticular disease and intestinal perforation (88) (**Figure 5**).

### Other patients’ features

**Figure 6:**
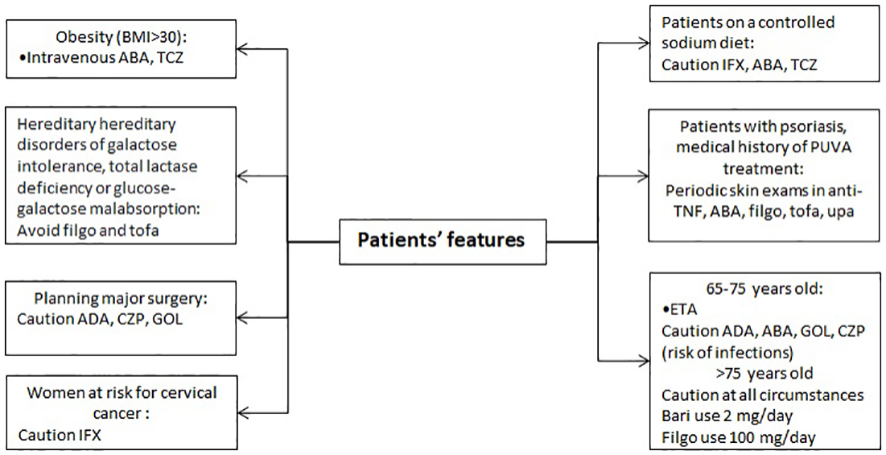
Therapeutic approach based on patient’s features.

Obesity, metabolic diseases and allergies can have an impact on drugs prescribability (**Figure 6**) (89). Obesity should be considered as a risk factor for DVT in patients treated with TOFA, FILGO and UPA (90). In obese patients (BMI > 30), intravenous route of administration should be preferred, using IFX, ABA, and TCZ (4, 6, 8). Indeed, it has been shown that obesity hampers the response to subcutaneous anti-TNF but not to ABA and TCZ (91). The possibility to administer higher doses in patients above 100 kg of body weight allows to reach satisfactory response rates in subjects treated with GOL, as confirmed by a recent Japanese study (92).

Treating a 65–75 years old patient directs the choice towards ETN as first-line agent (3) and draws attention on being cautious on the use of ADA, ABA, GOL and CZP (1, 2, 4, 6). Data on patients > 75 years old are limited to provide any strong suggestion. An increased rate of infections in this population is observed, thus caution should be used in all circumstances (93). BARI should be preferably given at 2 mg/day and filgotinib at 100 mg/day in patients ≥75 years old due to limited data on this population (94).

With regards to the planning of a major surgery, the product leaflet suggests a caution only for the use of ADA, IFN, CZP and GOL, especially due to the long half-life of these drugs (1, 2, 4). Nonetheless, it should be noted that controlling disease activity is a key factor for reducing the risk of major surgery in RA (95). Major surgery is also a risk factor for DVT to be considered for patients treated with TOFA, FILGO and UPA (90).

The data sheet of FILGO and TOFA suggests that patients with rare hereditary problems of galactose intolerance, total lactase deficiency or glucose-galactose malabsorption should avoid their use (11, 12).

Patients on a controlled sodium diet should be aware of the use of intravenous drugs. Despite IFX contains less than 1 mmol (23 mg) sodium per dose, i.e. essentially ‘sodium-free’, the drug is diluted using sodium chloride 9 mg/mL (0.9%) (5). Concerning ABA, the product contains 34.5 mg sodium in a maximum dose of 4 vials (8.625 mg sodium per vial), equivalent to 1.7% of the WHO recommended maximum daily intake for an adult (6). TCZ contains 1.17 mmol (or 26.55 mg) sodium per maximum dose of 1200 mg. Doses below 1025 mg contain less than 1 mmol (23 mg) sodium, i.e. practically “sodium free” (8). RTX contains sodium too, but no warnings are stated in the leaflet (7).

Before, and during treatment with anti-TNF, ABA, FILGO, TOFA, and UPA, periodic skin exams should be performed, especially in patients with massive use of immunosuppressive therapies or subjects with psoriasis who have medical history of psoralen and ultraviolet A (PUVA) treatment, to examine for the presence of a possible non-melanoma skin cancer (96). Melanoma and Merkel cell carcinoma have also been reported (1–6, 11–13, 96). This risk does not seem to be confirmed unequivocally in data from real-world (97). In women at risk for cervical cancer, IFX should be used with caution since an increased rate of this neoplasm has been reported in women with active RA treated with this drugs, compared to women with RA ever treated with biologics and the general population including women older than 60 years (5). Again, this risk was not confirmed in data from registry in patients with spondyloarthritis (98). An increased number of malignancies including lymphoma, compared to the general population, has been reported in clinical trials and post-marketing data in patients treated with TNF-blocking agents (1–5). There is not a definite conclusion whether other drugs may be associated with an increased risk of this complication that is *per se* a possible manifestation occurring at higher rates in patients with RA with very active and long-standing disease (99).

### Drugs administration and therapy features

**Figure 7:**
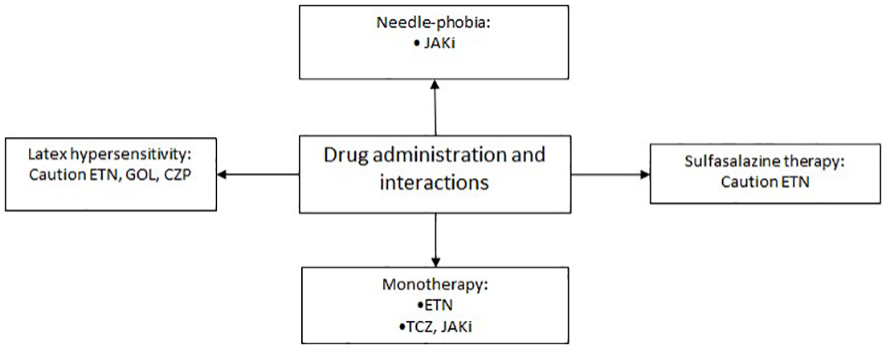
Therapeutic approach based on drug administration and interactions.

The choice of the drug must also consider the necessity/preference for route of administration, monotherapy or, if adding csDMARDs or GCs is appropriate (100–102), (**Figure 7**). Intravenous administration of a drug is related to a higher rate of adherence, on the other hand it is essential to recognize the quote of patients suffering from needle-phobia, without discrediting their situation (103, 104). In this case the best option results to be the oral administration of a JAKi (10, 11, 13, 105).

Patients who require monotherapy because intolerant or cannot assume csDMARDs should be started on ETN (3), leaving TCZ and JAKi (8, 10–13, 106) as second-line options. A recent meta-analysis showed that TCZ was superior to ADA, CZP, and GOL as monotherapy in RA. No differences among ETN and TCZ were found (106). Indeed, an evidence of no difference in efficacy of ETN in monotherapy vs combination therapy with csDMARDs was demonstrated, while both ADA and IFX demonstrated that co-medication with csDMARDs provides an additional advantage over anti-TNF monotherapy, in terms of either retention or DAS28 remission rate in RA (107). Of note, besides higher efficacy and longer persistence, the association of a b- or tsDMARD with a csDMARDs could predispose to a higher risk of infections and side effects. For instance, a higher number of side effects was observed in clinical trials of combination therapy of TOFA and MTX compared with TOFA monotherapy (108). Early reports (included in the product data sheet) suggested that the usage of combined therapy of ETN and sulfasalazine may require a certain grade of caution due to the possible effects on white blood cells count (18). However, more recent evidence did not find any safety issue, while combination therapy appears beneficial in patients with RA when compared with sulfasalazine alone (109). An assessment of risk/benefit ratio should be performed in all patients whether to choose mono or combo-therapy. In all circumstances, it is not recommended to use in combination with each other any of the drugs considered in this study or anakinra (15).

Allergies should be considered when choosing the best treatment strategy. The needle cap of the pre-filled syringe of ADA, ETN and GOL and the needle cap of the pen of GOL contain latex (dried natural rubber) which can cause hypersensitivity reactions (2–4).

### Laboratory abnormalities

RA treatment involves the use of drugs which can alter in many ways the profile of the patient’s blood test (110).

Since rare cases of pancytopenia, and very rare cases of aplastic anemia, some of which with fatal outcome, have been reported in patients treated with anti-TNF, caution should be used in patients receiving treatment with anti-TNF who have a history of blood dyscrasias (111).

Considering the red blood cells, in the case of moderate-severe anemia, the latest evidence suggests to avoid TOFA if the levels of hemoglobin are lower than 9 mg/dl and to avoid BARI, FILGO and UPA when they result to be lower than 8 mg/dl (10–13, 69).

Moving on an alteration of white cells, when we must deal with a neutropenia, it is suggested to choose an anti-TNF as first-line therapy: when this is not possible, not other drugs have specific evidence to be more adequate. However, with a neutrophil count of <2000/mmc, the use of TCZ and SAR is utterly contraindicated, since there are some studies which demonstrate the induction of a transient, dose-dependent neutropenia in patients treated with this drugs (8, 9, 112); with a neutropenia of <1000/mmc it is mandatory to avoid BARI, FILGO, TOFA and UPA (10–13). Likewise, with a situation of lymphocytopenia, the latest evidence and the drugs data sheets are about the same: avoiding TOFA with a count of <750/mmc and avoid BARI, FILGO and UPA with lymphocytes <500/mcc (10–13, 113).

Regarding thrombocytopenia, IL-6 inhibitors should be used with caution (114). In particular, if the thrombocytopenia is <150.000/ccm, SAR should be avoided (9); if it is <100.000/mmc, TCZ should be used with caution and the concomitant MTX dosage should be adjusted. TCZ should be avoided with platelet levels <50.000/mmc (8). It is recommended to evaluate the blood count before starting and then every 12 weeks when using b- or tsDMARds, after 4–8 weeks from drug commencement when using TCZ and SAR and only then every 12 weeks.

One of the most frequent adverse events after the administration of RTX is hypogammaglobulinemia (both IgM and IgG) (115, 116), which, even if usually asymptomatic, may expose fragile patients to an increased risk of infections even severe (7).

Several drugs which are used in common clinical practice (omeprazole, amiodarone, rifampicin, fluoxetine…) result to have an inductive or inhibitory function towards p450 cytochrome: this appears to be important to choose adequately the correct drug, so as to avoid possible interactions. Two phase 1, randomized, open-label, single sequence studies in 24 healthy subjects, have proven that the coadministration of Fluconazole and Ketoconazole (both inhibitor of CYP3A4) and TOFA, is likely to increase the systemic exposure to TOFA (117) and this may warrant dosage adjustments or restrictions. For this reason, in the clinical practice half dose should be considered when CYP3A4 and CYP2C19 inhibitors are simultaneously administrated (12); likewise, also the use of SAR and UPA should be managed with caution in the same situations (9, 13). In a clinical study the co-administration of UPA and ketoconazole has resulted in 70% and 75% increases in UPA Cmax and AUC, respectively. In another study, co-administration of UPA after multiple doses of rifampicin (potent CYP3A inducer) resulted in approximately 50% and 60% decrease of UPA Cmax and AUC, respectively. Moreover, since elevated levels IL-6 occurring in patients with RA can reduce CYP activity, thus increasing drug levels compared to subjects without RA, the blockade of IL-6 signaling can reverse the inhibitory effect of IL-6 and restore CYP activity, leading to altered drug concentrations.

Given its metabolism, in patients taking organic anion transporter 3 (OAT3) inhibitors with a strong inhibitory potential, such as probenecid, the recommended dose of BARI is 2 mg/daily.

TCZ, SAR, BARI, FILGO, TOFA and UPA should be used with caution in patients presenting an alteration of the lipid profile (8–10, 12, 13, 118). Patients should be managed according to international guidelines for hyperlipidemia and the lipid profile should be evaluated 12 weeks after initiation of BARI, FILGO and UPA (10, 11, 13), 8 weeks in TOFA treated patients (12), 4–8 weeks in TCZ and SAR treated patients and then every 6 months (8, 9).

It is not yet completely understood whether these drugs provoke also an increased cardiovascular risk due to modifications of the lipid profile (119). For instance, analysis of pooled data from TCZ-treated RA patients found that changes in lipid parameters were not statistically significantly associated with risk for on-treatment major adverse CV events (120), despite increases in TC, LDL-C, and TC/HDL-C ratio observed with TCZ treatment. Certainly, stratification of patients CV risk should be performed especially in patients treated with JAKi as abovementioned. The metabolism of BARI, FILGO and UPA which is prevalently renal, as well as it is partial for TOFA, suggests that these drugs can be harmful in the managing of a patient with RA who suffers from renal impaired function (121). Indeed, an estimated creatinine clearance (CrCl) of 30–60 ml/min suggests taking caution when administrating BARI. In this case, a dosage of 2 mg/day is recommended (10). In patients with a CrCl <30 ml/min also the usage of UPA requires caution, TOFA can be used at half dose, while Bari should be avoided (10, 12, 13). Concerning FILGO, this can be used at 100 mg/day in patients with CrCl 15–60 ml/min and should not be administered in patients with end-stage renal disease (CrCl<15 ml/min). IL-6 inhibitors turn out to be related to certain contraindications in patients with altered liver enzymes; likewise JAKi appear to have some contraindications too (10–13, 122–124). Concerning the latter, TOFA has a prevalent hepatic metabolism, while also UPA and FILGO and at a minor extent BARI are metabolized by the liver. Namely, a level 1–3x upper the limit of normal (ULN) of alanine aminotransferase (ALT) or aspartate aminotransferase (AST), implies to have caution on the use of TOFA, TCZ and SAR (8, 9, 12). MTX dosage (and that of any other concomitant csDMARD) should be adjusted, whenever possible, as first measure to counteract such increase of liver enzymes. In patients treated with TOFA, dose reduction of temporary suspension of the drug also proved useful in restoring normal levels of liver enzymes (12). If AST/ALT are > 3 to 5x ULN, this contraindicates the use of TCZ and SAR that should be suspended until liver enzymes are < 3x ULN (8, 9). Importantly, SAR treatment should be re-started at 150 mg every 2 weeks and increased at 200 mg every 2 weeks only when clinically possible. In the case of >5x ULN of ALT or AST, both TCZ and SAR treatment should be interrupted (8, 9). The levels of AST and ALT should be monitored 4–8 weeks after drug beginning and then every 3 months in SAR treated patients, in TCZ the monitoring should be performed every 4–8 weeks for the first 6 months of treatment and then every 3 months (8, 9).

Regarding the hepatic impairment expressed by the different levels of the Child-Pugh Score, a patient who belongs to the Child-Pugh B category should be treated with a half dose of TOFA (12), if this is the selected therapy; on a Child-Pugh C score, BARI, TOFA and UPA should be totally avoided (10, 12, 13). FILGO was not studied in patients with severe liver dysfunction (Child-Pugh C) thus its usage is not recommended in this condition (11).

**Figure 8:**
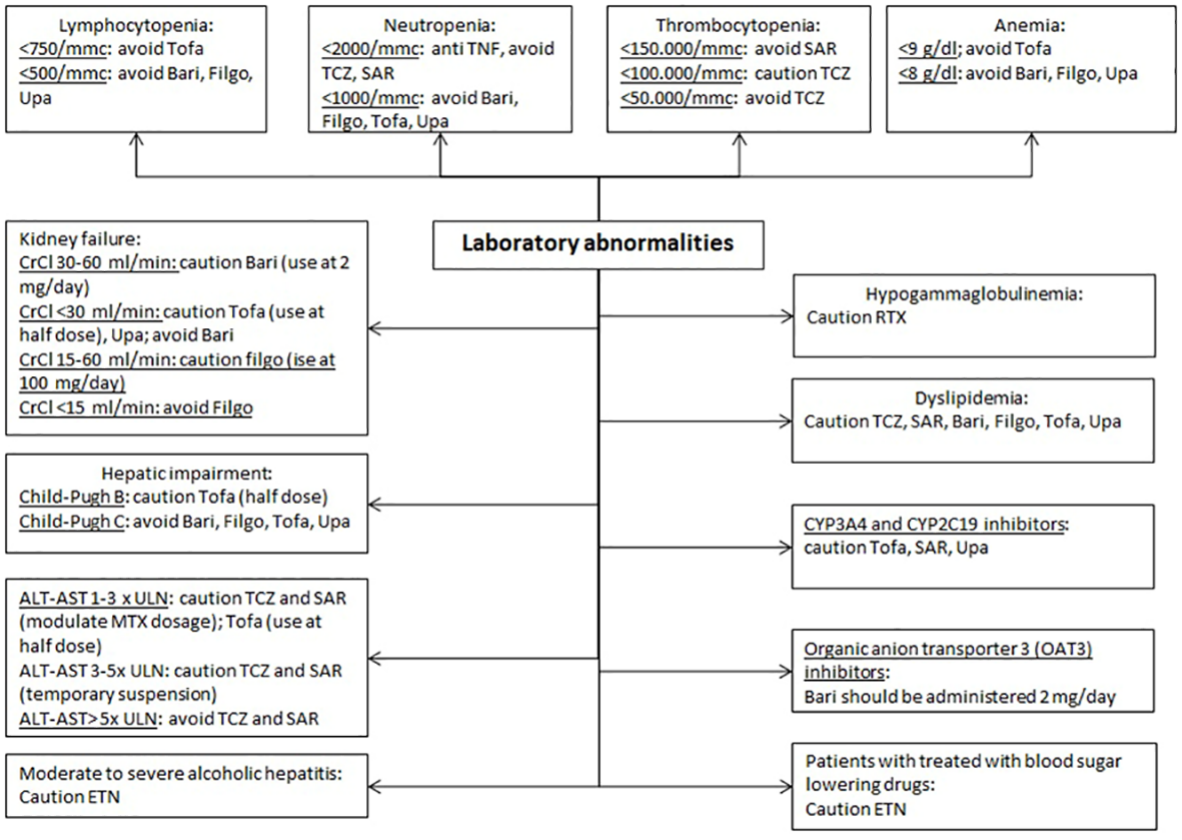
Therapeutic approach based on laboratory abnormalities.

Patients with moderate to severe alcoholic hepatitis should be treated with caution with ETN. Also, there are reports of hypoglycemia in patients being treated for diabetes after initiating ETN therapy, thus requiring lowering of the medicines for diabetes (125) (**Figure 8**). ETN should be used with caution in patients with advanced or uncontrolled diabetes due to the risk of infection in these patients. This warning may be extended to all the mentioned drugs (126).

Interference with some coagulation assays, specifically partial thromboplastin time test for Lupus Anticoagulant and the automated partial thromboplastin time, has been observed in patients treated with CZP that may cause falsely elevated partial thromboplastin time assay results activated in patients without abnormalities of the coagulation process. Abnormal coagulation tests results should be carefully evaluated in patients receiving CZP (127).

## Conclusions

Recent EULAR recommendations provide the most robust algorithm and treatment strategy for patients with RA with synthetic and biological DMARDs. However, some specific situations which are accounted in product leaflets were not considered or mentioned.

Thus, we provided decisional trees as the result of the combination of patients’ features and available product leaflets in Italy. This is the first study performed with this innovative approach, and we hope that the information gathered from the product leaflet data sheets of the currently approved drugs for the treatment of RA with most recent advances from the literature will inform rheumatologists, health professionals, patients, regulators, payers, and other stakeholders on the current in-label approach to these patients.

Product leaflets are useful to inform physicians as well as patients as they summarize most of the information on each drug. However, they may contain data that should be carefully considered at the light of current clinical practice. Indeed, in some circumstances an update (such in the case of ETN and sulfasalazine combination) or a revision (as per the approach to pregnant and lactating patients) would better fit with the most recent evidence from the literature. New findings will surely occur over-time and we expect a need for an update of this material in the next 3–5 years.

## Data availability statement

The original contributions presented in the study are included in the article/supplementary material. Further inquiries can be directed to the corresponding author.

## Author contributions

CP: Conceptualization, Data curation, Supervision, Writing – original draft, Writing – review & editing. ACa: Data curation, Methodology, Resources, Writing – original draft, Writing – review & editing. GC: Conceptualization, Data curation, Methodology, Validation, Writing – review & editing. SC: Investigation, Resources, Writing – review & editing. LB: Data curation, Writing – review & editing. RP: Data curation, Methodology, Writing – review & editing. FT: Investigation, Writing – review & editing. ACo: Data curation, Methodology, Writing – review & editing. RG: Supervision, Writing – review & editing. EB: Methodology, Project administration, Supervision, Validation, Writing – review & editing.
